# Pretransplant CMV-IgG Titers and DNAemia Are Associated With CMV Infections Post Allogeneic Hematopoietic Cell Transplant With or Without Letermovir

**DOI:** 10.1093/ofid/ofag068

**Published:** 2026-02-16

**Authors:** Léna Royston, Ming Zou, Federico Simonetta, Manuel Schibler, Sabine Yerly, Amandine Pradier, Federica Giannotti, Anne-Claire Mamez, Sarah Morin, Christian Van Delden, Laurent Kaiser, Yves Chalandon, Stavroula Masouridi-Levrat, Dionysios Neofytos

**Affiliations:** Division of Infectious Diseases, University Hospital of Geneva, Geneva, Switzerland; University Hospital Basel, Basel, Switzerland; Division of Hematology, Bone Marrow Transplant Unit, University Hospital of Geneva, Geneva, Switzerland; Translational Research Center for Oncohematology, Department of Medicine, Faculty of Medicine, University of Geneva, Geneva, Switzerland; Division of Infectious Diseases, University Hospital of Geneva, Geneva, Switzerland; Virology Laboratory, University Hospital of Geneva, Geneva, Switzerland; Virology Laboratory, University Hospital of Geneva, Geneva, Switzerland; Division of Hematology, Bone Marrow Transplant Unit, University Hospital of Geneva, Geneva, Switzerland; Translational Research Center for Oncohematology, Department of Medicine, Faculty of Medicine, University of Geneva, Geneva, Switzerland; Division of Hematology, Bone Marrow Transplant Unit, University Hospital of Geneva, Geneva, Switzerland; Division of Hematology, Bone Marrow Transplant Unit, University Hospital of Geneva, Geneva, Switzerland; Division of Hematology, Bone Marrow Transplant Unit, University Hospital of Geneva, Geneva, Switzerland; Translational Research Center for Oncohematology, Department of Medicine, Faculty of Medicine, University of Geneva, Geneva, Switzerland; Division of Infectious Diseases, University Hospital of Geneva, Geneva, Switzerland; Division of Infectious Diseases, University Hospital of Geneva, Geneva, Switzerland; Division of Hematology, Bone Marrow Transplant Unit, University Hospital of Geneva, Geneva, Switzerland; Division of Hematology, Bone Marrow Transplant Unit, University Hospital of Geneva, Geneva, Switzerland; Division of Infectious Diseases, University Hospital of Geneva, Geneva, Switzerland

**Keywords:** CMV (cytomegalovirus), DNAemia, hematopoietic cell transplantation, letermovir, serology

## Abstract

**Background:**

The impact of pretransplant cytomegalovirus (CMV) serology and DNAemia on posttransplant clinically significant CMV infections (csCMVi) in allogeneic hematopoietic cell transplant recipients (allo-HCTR) is poorly described.

**Methods:**

We performed a single-center cohort study of adult allo-HCTR (16 November 2015–31 December 2023). Letermovir prophylaxis was administered after 01 May 2019 during 100 days (d) posttransplant in CMV-seropositive patients (R+). We investigated associations of pretransplant CMV-IgG-titers and CMV-DNAemia with posttransplant csCMVi and CMV-DNAemia during 6 months posttransplant. In CMVR+, CMV-IgG-titers were classified as “low” (<40 IU/mL) or “high” (≥40 IU/mL). Quantitative CMV-PCR was performed pre-HCT, weekly for 3 months and as clinically indicated.

**Results:**

Among the 485 patients included, 209 and 276 underwent a first allo-HCT in the pre- and postletermovir periods, respectively; 314/485 (64.7%) patients were CMVR + . Patients with high CMV-IgG-titers had a higher incidence of csCMVi by d180 posttransplant (47.6%, 95% CI: 41.4–53.6) than those with low CMV-IgG-titers (22.5%, 95% CI: 11.5–35.7) and CMVR- (3.6%, 95% CI: 1.5–7.2, *P* < .001), and higher incidence of any CMV-DNAemia (*P* < .001). Overall, 61/485 (12.6%) patients had detectable/quantifiable pretransplant CMV-DNAemia, with higher posttransplant incidence of csCMVi by d180 (61.3%, 95% CI: 47.6–72.4) compared to those with undetectable pretransplant CMV-DNAemia (26.9%, 95% CI: 22.3–31.6, *P* < .001) and higher incidence of any CMV-DNAemia (*P* < .001). Pretransplant high CMV-IgG-titers (aHR: 14.18, *P* < .001) and CMV-DNAemia (aHR: 2.43, *P* < .001) were strong predictors of posttransplant csCMVi.

**Conclusions:**

Pretransplant high CMV-IgG-titers and detectable/quantifiable DNAemia were associated with higher posttransplant csCMVi/CMV-DNAemia incidence, including in the letermovir era. Additional studies on the clinical utility of baseline pretransplant CMV burden in CMV prophylaxis stratification algorithms are needed.

## Teaser

Pretransplant high CMV-IgG-titers and detectable CMV-DNAemia can predict clinically significant CMV infections posthematopoietic cell transplantation, including in patients receiving letermovir prophylaxis. Using those parameters in CMV risk stratification algorithms could help to tailor antiviral prophylaxis.

Despite the efficacy of letermovir prophylaxis in reducing the incidence of clinically significant cytomegalovirus infections (csCMVi), CMV remains a significant burden in allogeneic hematopoietic cell transplant (allo-HCT) recipients [1, 2]. Both breakthrough and postletermovir rebound csCMVi occur, requiring treatment with other antiviral drugs with inherent toxicities, and leading to significant morbidity and prolonged hospitalizations [3, 4]. Furthermore, letermovir prophylaxis has been associated with delayed anti-CMV immune reconstitution and remains very expensive [1, 5]. It is thus essential to better stratify the risk of csCMVi, to optimize post-allo-HCT management based on individual predisposition to infection. While CMV-specific cell-mediated immunity (CMI) appears to be a promising tool, the clinical significance of pretransplant CMV-IgG-titers and CMV-DNAemia to predict posttransplant csCMVi requires further investigation [6, 7]. Zamora *et al* recently identified pretransplant CMV reactivation as a risk factor for posttransplant CMV reactivation and disease [8]. We have also previously reported that CMV-DNAemia before allo-HCT may be associated with posttransplant csCMVi (OR 7.1, *P* = .05) [9]. In addition, data from 5 centers suggested associations between pretransplant CMV-IgG levels and posttransplant CMV reactivation [10]. We hypothesized that pretransplant CMV-IgG-titers and CMV-DNAemia in the recipient may indicate a higher burden of subclinical CMV activity of the viral latent reservoir leading to more frequent posttransplant csCMVi. In order to study pretransplant virological variables as potential posttransplant csCMVi predictors, we performed a single-center cohort study to describe the association between pretransplant CMV-IgG-titers and CMV-DNAemia with the incidence of csCMVi by d180 posttransplant.

## METHODS

### Study Design

This is a single-center cohort study including all consecutive adult allo-HCT recipients transplanted at our institution from 16 November 2015 to 31 December 2023. In case of multiple allo-HCT, only the first transplantation administered during the study period was included. The study period was selected based on the fact that different CMV-IgG serology assays were used at our institution before 16 November 2015. The study was approved by local ethics committee (2020-00059, 2020-02120). All patients included had signed an informed consent form for data utilization for clinical research.

### Study Objectives

The primary objective was to describe incidence of CMV events between d0-d180 posttransplant, based on pretransplant CMV-IgG-titers and CMV-DNAemia. As CMV quantitative PCR assays and preemptive treatment initiation thresholds changed during the study period, 3 virological outcomes were studied to describe the overall posttransplant CMV events: (1) csCMVi defined based on institutional guidelines during the study period, (2) any (detectable/quantifiable) CMV-DNAemia, and (3) the number of positive (detectable/quantifiable) CMV PCR results. Secondary objectives included identification of csCMVi predictors and associations of pretransplant CMV-IgG-titers/CMV-DNAemia with nonvirological clinical outcomes, including all-cause mortality, hematological relapse, and grade ≥ 2 acute graft-versus-host-disease (GvHD) by 1-year posttransplant.

### Laboratory Procedures

Since 16 November 2015, CMV-IgG-titers are measured using Elecsys® CMV-IgG (Roche Diagnostics, Switzerland), with a negativity cutoff of <0.6 IU/mL, indeterminate results between ≥0.6 and ≤3 IU/mL, and positive results >3 IU/mL (range of quantification, 0–500 IU/mL) as per manufacturer's recommendations. Plasma CMV quantitative PCR (qPCR) was performed with the COBAS CMV for Cobas 6800 test (Roche Diagnostics, IN) since 16 May 2018 (limit of detection (LOD) and quantification (LOQ): 21 and 26 IU/mL, respectively), and previously with the COBAS AmpliPrep/COBAS TaqMan CMV test (Roche Diagnostics, IN) (LOD and LOQ of 56 and 137 IU/mL, respectively) [1].

### Institutional Procedures

All allo-HCT recipients have transplant infectious disease (TID) consultation within approximately 4 weeks before transplant, with CMV serology and DNAemia performed at the time of that visit [11]. Consistent with our institutional algorithm and recent guidelines by the European Conference on Infections in Leukemia-10, patients with positive CMV serology at their pretransplant evaluation based on indeterminate (0.6–3 IU/mL) or low (>3 to <50 IU/mL) positive CMV-IgG-titers, with negative CMV-DNAmia at the same time, but with negative CMV serology at the time of hematologic malignancy diagnosis are reclassified from CMV recipient (R)+ to CMV R- [11–13]. As previously described the cutoff of 50 IU/mL was arbitrarily chosen based on our clinical observations and experience [12]. Plasma CMV qPCR is performed once weekly during 3 months and approximately every other week or as clinically indicated thereafter [1]. Letermovir primary CMV prophylaxis was introduced on 01 May 2019 to all CMV donor (D)-/R + patients from posttransplant day (d)1 to d100 until 31 December 2020, and to all CMVR + allo-HCT recipients starting 01 January 2021 [1, 4]. CMVR + with early (before d100 posttransplant) grade ≥2 acute GvHD requiring corticosteroid treatment ≥1 mg/kg/day receive primary letermovir prophylaxis until tapering to <10 mg/day of prednisone [1, 4]. Preemptive treatment for csCMVi was initiated for CMV-DNAemia ≥ 150 IU/mL until 31 December 2022 and thereafter at ≥500 IU/mL in patients on letermovir or ≥150 IU/mL in patients not receiving prophylaxis.

### Study Definitions

**Figure 1.**
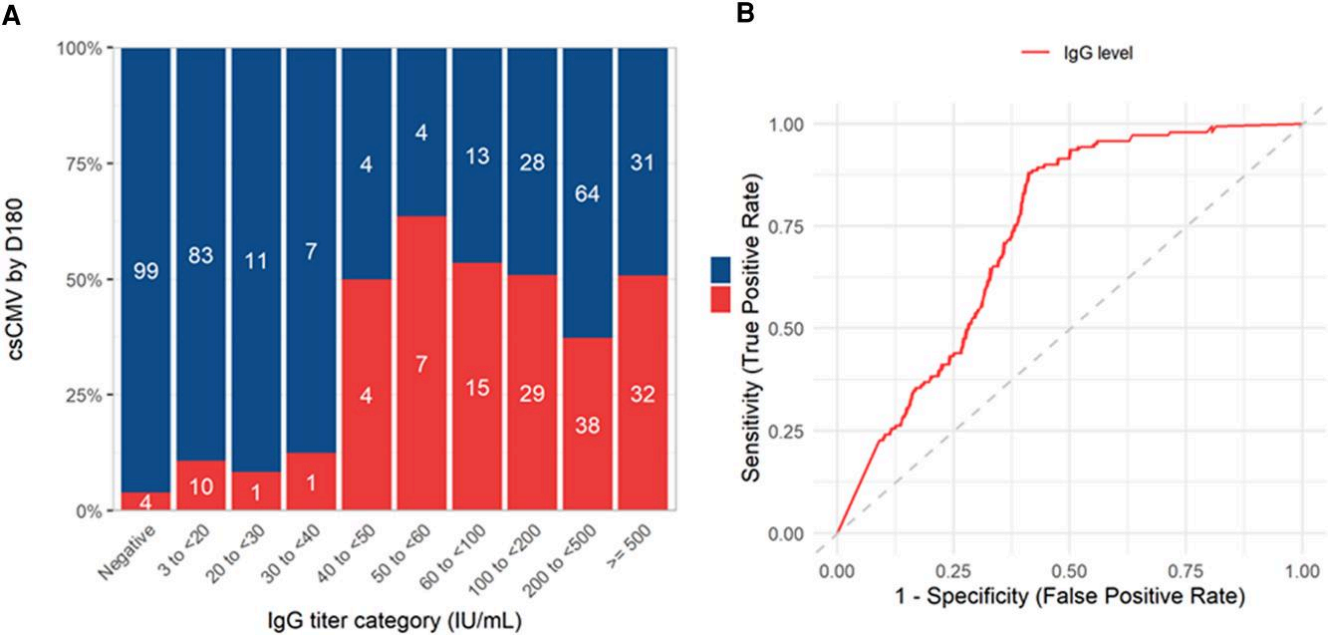
Proportion of patients experiencing clinically significant CMV infection by day 180 posttransplant based on pretransplant CMV-IgG-titers (*A*). Clinically significant CMV infection was recorded as defined during the time period. Receiver Operating Characteristic (ROC) curve analysis to evaluate the IgG levels in distinguishing patients with higher risk for csCMVi (*B*): the area under the curve (AUC) was 0.73 (95% CI: .69–.78) and the optimal cutoff determined by Youden's index was at 44 IU/mL, with a sensitivity and specificity of 88% and 59%, respectively.

Clinically significant CMV infection was defined as CMV end-organ disease and/or CMV-DNAemia leading to administration of preemptive therapy, based on international consensus definitions. Based on our institutional protocols, preemptive CMV therapy was administrated in patients with CMV-DNAemia ≥ 150 IU/mL from 16 November 2015 to 31 December 2022 and ≥500 IU/mL or ≥150 IU/mL in patients receiving or not letermovir prophylaxis, respectively, thereafter. Although most patients have pretransplant TID consultation within 4 weeks prior to their transplant, we accepted as pretransplant CMV-IgG-titers the closest value to the transplant from 24-months before HCT until d0. To identify a cutoff of pretransplant CMV-IgG positivity above which posttransplant CMV-DNAemia was more frequent, we plotted posttransplant csCMVi across different pretransplant CMV-IgG values (Figure 1*A*). A cutoff of pretransplant CMV-IgG titer >40 IU/mL appeared to be closely related to posttransplant csCMVi. In addition, a receiver operating characteristic curve analysis was performed to evaluate the IgG levels in distinguishing patients with higher risk for csCMVi (Figure 1*B*). The area under the curve was 0.73 (95% CI: 0.69–0.78) and the optimal cutoff determined by Youden's index was at 44 IU/mL, with 88% of sensitivity and 59% of specificity. Based on these observations, a cutoff of 40 IU/mL was ultimately selected to define pretransplant CMV-IgG-titers >0.6–40 and >40 IU/mL as “low” and “high,” respectively. Pretransplant CMV-DNAemia was defined as any positive plasma CMV PCR (detectable or quantifiable with the qPCR assay available at the time) since d-60 pretransplant until d0 of HCT. During the study period, there was no standardized approach for the management of pretransplant CMV-DNAemia at our institution. The study period was divided in preletermovir and postletermovir periods, before and after 01 May 2019, respectively.

### Statistical Analyses

Data were analyzed descriptively to provide counts, percentages, median values, interquartile ranges (IQR), and 95% CI as appropriate. Cumulative incidences were calculated for post-HCT csCMVi/CMV-DNAemia by d180, with death and loss to follow-up being censored. Kaplan–Meier analyses of all-cause mortality, hematological relapse, and acute GvHD ≥ grade 2 were performed for the first year post-HCT. Log-rank and Peto-Peto tests were used to assess differences in cumulative incidences and Kaplan–Meier analyses. Wilconxon paired test was used to assess difference of numbers of positive CMV PCR tests. Cox proportional hazards regression was used to estimate the association of pre-HCT CMV-IgG titer and CMV-DNAemia, among patients and HCT characteristics, with the post-HCT csCMVi outcomes. Statistical significance was assessed at a 2-sided 0.05 level for all analyses. Analyses were conducted with R v4.3.2 and reported with RepTik v2.5.

## RESULTS

### Patient Population

Overall, 485 patients received a first allo-HCT during the study period: 209 (43%) and 276 (57%) in the pre- and postletermovir period. Patient characteristics were similar between the 2 periods (Table 1). All patients had a pretransplant CMV-IgG performed (median 30d pre-HCT; IQR: 43, 22): 416/485 (86%) and 69/485 (14%) patients were initially assessed as CMVR + and R-, respectively. Amongst CMVR+, there were 34/416 (8%) patients with indeterminate (0.6–3 IU/mL) pretransplant CMV-IgG-titers. Furthermore and using a prior published algorithm (negative CMV serology at malignancy diagnosis with positive pretransplant CMV-IgG-titers < 50 IU/mL and undetectable CMV-DNAemia), 102/416 (25%) patients (46 and 56 patients, in the pre- and postletermovir periods, respectively) were reclassified from CMVR + to R- (Supplementary Figure 1) [12]. Among patients with low-positive CMV-IgG-titers, 9 could not be reclassified due to missing CMV serology at diagnosis, and 3 were not reclassified by the clinician in charge despite fulfilling the algorithm criteria. In total, 314/485 (65%) patients were considered CMVR+, including 137/209 (66%) and 177/276 (64%) in the pre- and postletermovir periods, respectively. Pretransplant CMV PCR results were available in 421/485 (86.8%) patients: 206/209 (99%) and 212/276 (77%) in the pre- and postletermovir periods, respectively. Sixty-one patients (13%) had positive pretransplant plasma CMV-DNAemia, with 24/61 (39%) detectable and 37/61 (61%) quantifiable, and with a mean CMV-DNAemia of 1677 IU/mL (range 21–95′600 IU/mL). Overall, 141/485 patients (29%) experienced a csCMVi by d180 posttransplant, including 88/209 (42%) and 53/276 (19%) in the pre- and postletermovir periods, respectively. Similarly, 263/485 patients (54%) had ≥1 detectable DNAemia by d180 posttransplant, including 115/209 (55%) and 148/276 (54%) in the pre- and postletermovir periods, respectively. In total, 8 patients had CMV end-organ disease, 7 and 1 in the pre- and postletermovir period respectively: 5 had biopsy-proven disease (pneumonia, n = 2, hepatitis, n = 1, colitis, n = 1, and encephalitis, n = 2) and 3 were classified as probable CMV disease (pneumonia, n = 2, and retinitis, n = 1).

**Table 1.** Patient Baseline Characteristics

	Overall (%)	Preletermovir Period (%)	Postletermovir Period (%)
**Total number of patients**	**485**	**209**	**276**
**Demographics**			
Median age at HCT, year (IQR)	57 (47; 65)	56 (48; 63)	57 (45; 66)
Sex, Male	311 (64)	133 (64)	178 (64)
**Primary disease**			
AML or MDS	293 (60)	130 (62)	163 (59)
Other**^a^**	192 (40)	79 (38)	113 (41)
**Pretransplant CMV-IgG**			
High titers	267 (55)	109 (52)	158 (57)
Low titers	47 (10)	28 (13)	19 (7)
Negative	171 (35)	72 (34)	99 (36)
**Pretransplant CMV PCR**			
Positive	61 (13)	25 (12)	36 (13)
Undetectable	360 (74)	181 (87)	179 (65)
Missing	65 (13)	3 (1)	62 (21)
**Median Karnofsky score (IQR)**	90 (90; 100)	90 (90; 100)	100 (90; 100)
**Conditioning regimen**			
Myeloablative	105 (22)	49 (23)	56 (20)
Reduced-intensity	380 (78)	160 (77)	220 (80)
**Donor type**			
HLA-matched related	106 (22)	49 (23)	57 (21)
HLA-matched unrelated	228 (47)	104 (50)	124 (45)
HLA-mismatched related	125 (26)	44 (21)	81 (29)
HLA-mismatched unrelated	26 (5)	12 (6)	14 (5)
**Donor, CMV serology positive**	261 (54)	109 (52)	152 (55)
**Stem cell source**			
BM	50 (10)	36 (17)	14 (5)
PBSC	435 (90)	173 (83)	262 (95)
**Risk of relapse**			
Low	24 (5)	9 (4)	15 (5)
Intermediate	331 (68)	138 (66)	193 (70)
High	104 (21)	52 (25)	52 (19)
Very high	18 (4)	9 (4)	9 (3)

HCT, hematopoietic cell transplant; AML, acute myeloid leukemia; MDS, myelodysplastic syndrome, HLA, human leukocyte antigen; GvHD, graft vs host disease, CMV, cytomegalovirus, IQR, interquartile range, BM, bone marrow, PBSC, peripheral blood stem cells.

^a^Other included the following: lymphoma (59), acute lymphoid leukemia (46), chronic myeloid leukemia (16), myeloproliferative disorder (19), chronic lymphoid leukemia (6), hemoglobinopathy (5), aplastic anemia (2), and inborn errors (1).

### Pretransplant CMV-IgG-titers and Posttransplant CMV Infection

**Figure 2.**
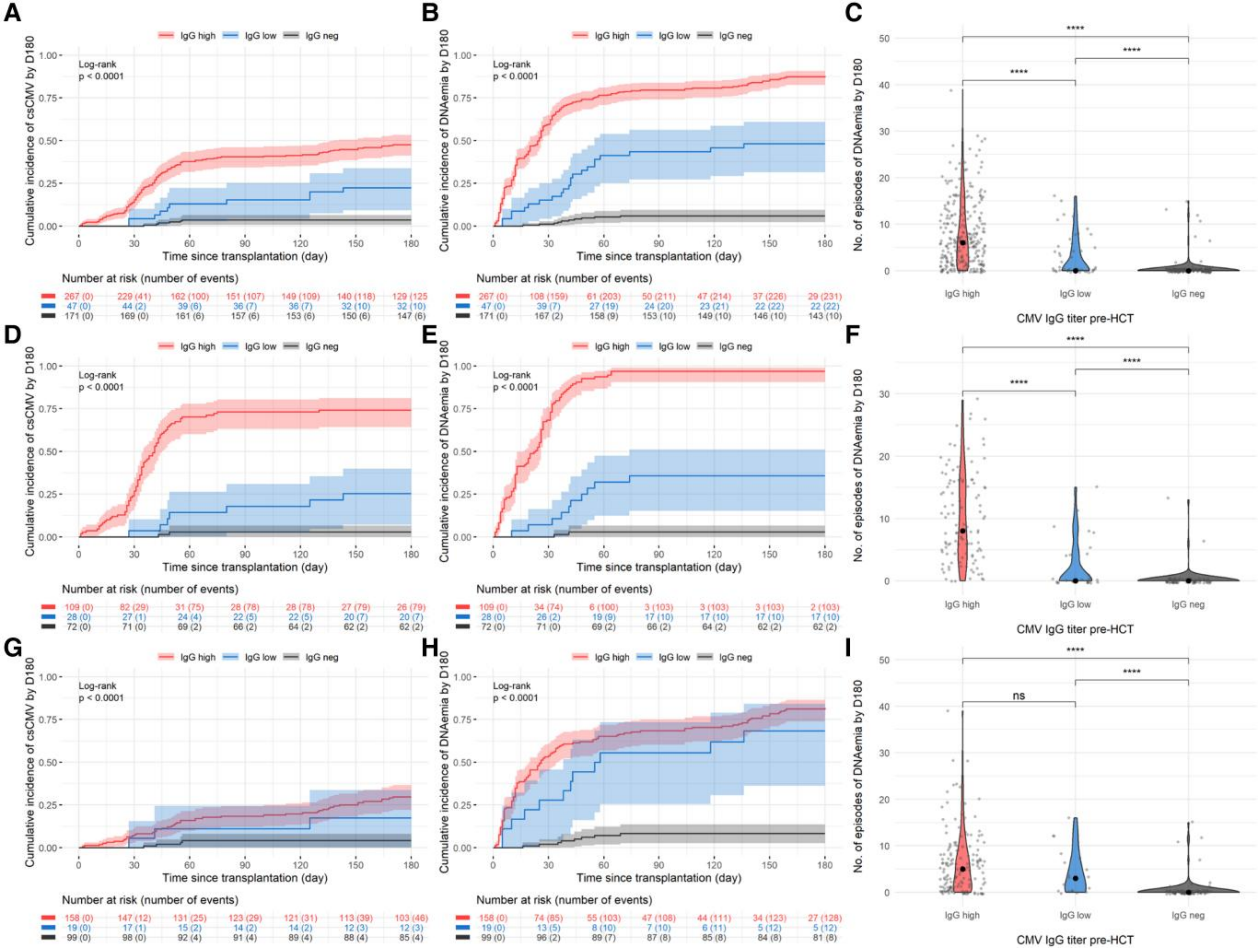
CMV burden by day 180 stratified by pretransplant CMV-IgG-titers in the whole cohort (*A–C*), preletermovir (*D–F*) and postletermovir period (*H–I*). Pretransplant CMV-IgG-titers have been classified as high (positive and IgG ≥40 IU/mL, n = 267), low (positive and IgG <40 IU/mL n = 47), or negative (n = 171). *A*, *D*, *G*, Cumulative incidence of the first clinically significant CMV (csCMV) infection (≥150 IU/mL until 31 December 2022 or ≥500 IU/mL thereafter) in the high, low and negative IgG groups, respectively (detailed in Supplementary Table 1). *B*, *E*, *H*, Cumulative incidence of the first detected DNAemia (≥56 IU/mL until 16 May 2018 and ≥21 IU/mL thereafter) in the high, low and negative IgG groups, respectively. *C*, *F*, *I*. Comparison of the number of CMV-DNAemia episodes detected by d180. Abbreviation: Ns, nonsignificant, ****P < .0001.

There were 171/485 (35%), 47/485 (10%) and 267/485 (55%) patients with negative, low, and high positive CMV-IgG-titers, respectively (Table 1). Overall, patients with high CMV-IgG-titers had a higher incidence of csCMVi posttransplant (cumulative incidence: 47.6%, 95% CI: 41.4–53.6, Supplementary Table 1) than the low CMV-IgG titer (22.5%, 95% CI: 11.5–35.7) and CMVR- group (3.6%, 95% CI: 1.5–7.2, *P* < .001, Figure 2*A*). Similarly, incidence of any (detectable/quantifiable) CMV-DNAemia during the first 6 months posttransplant was significantly higher in the high CMV-IgG titer group (87.8%, 95% CI: 83.0–91.3), compared with patients with low CMV-IgG-titers (48.3%, 95% CI: 33.0–62.0) and CMV R- (6.0%, 95% CI: 3.0–10.3, *P* < .001, Figure 2*B*). The number of positive CMV PCR tests by d180 posttransplant was significantly higher in patients with high versus low CMV-IgG-titers and CMVR- patients (*P* < .001; Figure 2*C*). Results were similar and remained significant between the high, low CMV-IgG titer and CMVR- groups for all 3 different virologic outcomes in the preletermovir (*P* < .001, Figure 2*D*-*F*) and postletermovir study periods (*P* < .001, Figure 2*G***-***I*).

### Pretransplant CMV-DNAemia and Posttransplant CMV Infection

**Figure 3.**
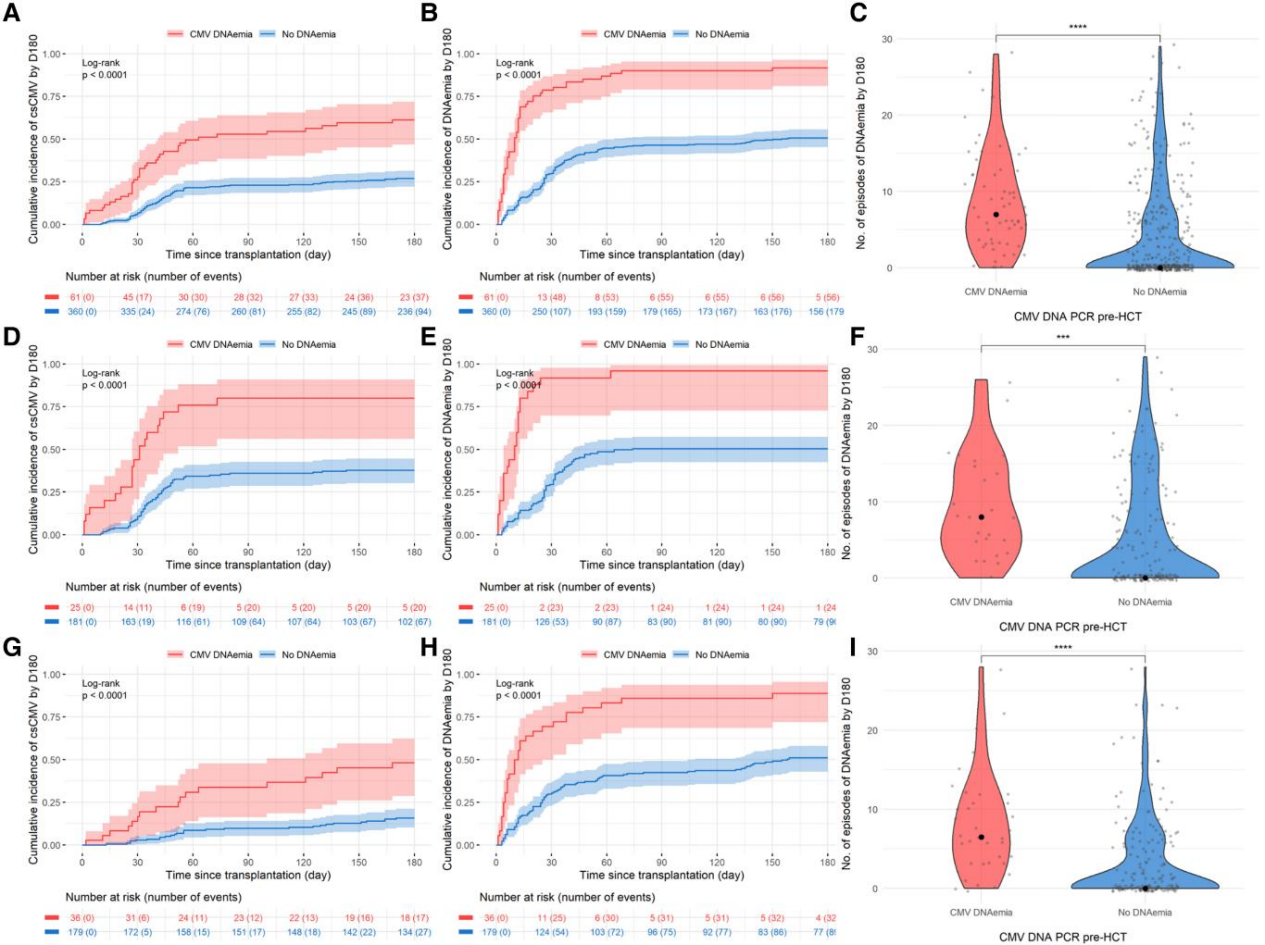
CMV burden by day 180 stratified by pretransplant CMV-DNAemia in the whole cohort (*A–C*), pre- (*D–F*) and postletermovir study period (*H–I*). Pretransplant DNAemia refers to the detection of at least 1 positive (detectable or quantifiable) CMV PCR from d-60 before the transplant until d0 (n = 61), and no DNAemia as undetectable CMV PCR (n = 360). *A*, *D*, *G*, Cumulative incidence of the first clinically significant CMV (csCMV) infection (≥150 IU/mL until 31 December 2022 or ≥500 IU/mL thereafter) in the DNAemia detectable and nondetectable groups, respectively (detailed in Supplementary Table 1). *B*, *E*, *H*, Cumulative incidence of the first detected DNAemia (≥56 IU/mL until 16 May 2018 and ≥21 IU/mL thereafter) in the DNAemia detectable and nondetectable groups, respectively. *C*, *F*, *I*, Comparison of the number of CMV-DNAemia episodes detected by d180. Abbreviation: Ns, nonsignificant, ***P < .001, ****P < .0001.

Overall, 61/485 patients (13%) had a positive CMV PCR before HCT: 25/209 (12%) and 36/276 (13%) in the pre- and postletermovir periods, respectively (Table 1). Patients with pretransplant CMV-DNAemia had a significantly higher incidence of csCMVi (61.3%, 95% CI: 47.6–72.4, Supplementary Table 1) compared to patients with undetectable pretransplant CMV PCR (26.9%, 95% CI: 22.3–31.6, *P* < .001, Figure 3*A*). Similarly, incidence of any (detectable/quantifiable) CMV-DNAemia during the first 6 months posttransplant was significantly higher in the group with pretransplant CMV-DNAemia (*P* < .001, Figure 3*B*). A higher number of positive CMV PCR tests was observed in patients with pretransplant CMV-DNAemia versus not (*P* < .001, Figure 3*C*). Those differences remained significant in the pre- (*P* < .001, Figure 3*D*-*F*) and postletermovir study periods (*P* < .001, Figure 3*G*-*I*).

### Combined Pretransplant CMV-IgG-titers and CMV-DNAemia and Posttransplant CMV Infection

**Figure 4.**
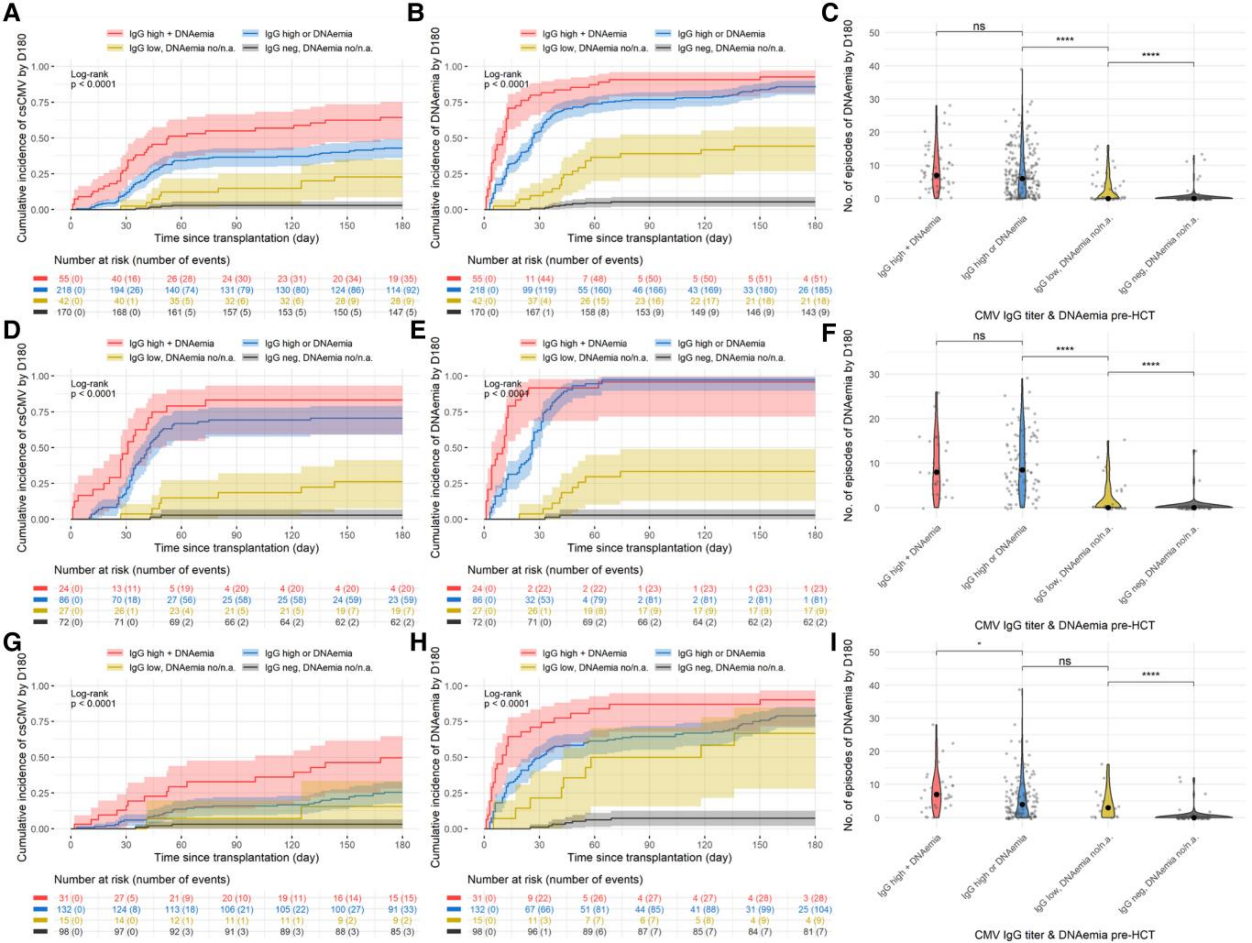
CMV burden by day 180 stratified by the combination of pretransplant CMV-IgG-titers and CMV-DNAemia in the whole cohort (*A–C*), pre- (*D–F*) and postletermovir study period (*H–I*). Four groups are compared, patients with: both high pretransplant CMV-IgG-titers and pretransplant CMV-DNAemia (n = 55), either high pretransplant CMV-IgG-titers or pretransplant CMV-DNAemia (n = 218), both low pretransplant CMV-IgG-titers and undetectable (or not performed) pretransplant CMV PCR (n = 42), negative CMV-IgG and undetectable DNAemia pretransplant (n = 170). *A*, *D*, *G*, Cumulative incidence of the first clinically significant CMV (csCMV) infection (≥150 IU/mL until 31 December 2022 or ≥500 IU/mL thereafter) in the high IgG/DNAemia, high IgG or DNAemia, low IgG/undetectable DNAemia and negative IgG/undetectable DNAemia, respectively (detailed in Supplementary Table 1). *B*, *E*, *H*, Cumulative incidence of the first detected DNAemia (≥56 IU/mL until 16 May 2018 and ≥21 IU/mL thereafter) in the high IgG/DNAemia, high IgG or DNAemia, low IgG/undetectable DNAemia and negative IgG/undetectable DNAemia, respectively. *C*, *F*, *I*, Comparison of the number of CMV-DNAemia episodes detected by d180. Abbreviation: Ns, nonsignificant, **P < .01, ****P < .0001.

We combined CMV-IgG-titers and CMV-DNAemia pretransplant in 3 groups of CMVR + patients: patients with both high pretransplant CMV-IgG-titers and CMV-DNAemia (55/485, 11%), either high pretransplant CMV-IgG-titers or CMV-DNAemia (218/485, 45%), and both low pretransplant CMV-IgG-titers and undetectable (or not performed) CMV PCR (42/485, 9%). Those groups were compared to patients with both negative CMV-IgG and undetectable CMV-DNAemia (170/485, 35%). Patients with both high CMV-IgG and CMV-DNAemia had a higher incidence of csCMVi (62.1%, 95% CI: 47.9–73.5) compared to the group with either high CMV-IgG-titers or DNAemia (42.6%, 95% CI: 36.0–49.0) or the group with low CMV-IgG-titers and no DNAemia (20.3%, 95% CI: 9.9–33.3, *P* < .001, Figure 4*A*). Results were similar for any positive CMV-DNAemia by d180 (Figure 4*B*, *P* < .001) and the number of positive CMV PCR tests (Figure 4*C*, *P* < .001). These differences were significant in the pre- (*P* < .001, Figure 4*D*-*F*) and postletermovir periods (*P* < .001, Figure 4*G*-*I*).

### Predictors of Posttransplant csCMVi

**Figure 5.**
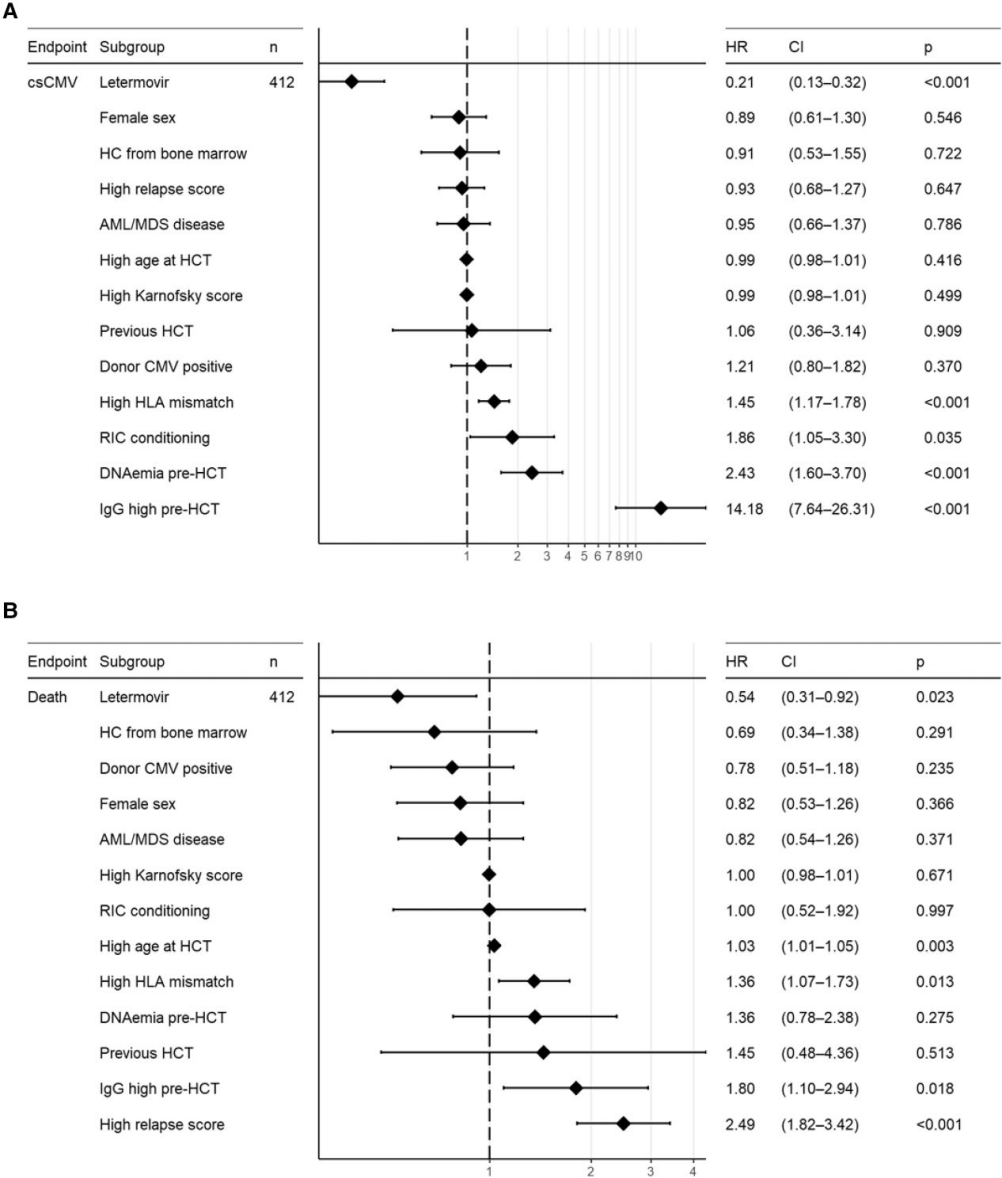
Risk factors for csCMVi by day 180 posttransplant using Cox regression analysis (*A*). Predictors of mortality at 1-y posttransplant using Cox regression analysis (*B*). Abbreviations: HR, hazard ratio; CI, confidence interval.

Patient/allo-HCT characteristics, and pretransplant CMV-IgG-titers and DNAemia were evaluated as potential predictors of csCMVi by d180 posttransplant using multivariable Cox regression analysis (Figure 5*A*). High pretransplant CMV-IgG-titers (adjusted hazard ratio, aHR 14.18, 95% CI: 7.64–26.31, *P* < .001), pretransplant CMV-DNAemia (aHR 2.43, 95% CI: 1.60–3.30, *P* < .001), and HLA-mismatch (aHR 1.45, 95% CI: 1.17–1.78, *P* < .001) were associated with posttransplant csCMVi. In contrast, letermovir administration was associated with a significantly lower risk of csCMVi (aHR 0.21, 95% CI: .13–.32, *P* < .001).

### Pretransplant CMV-IgG-titers or CMV-DNAemia and Posttransplant Nonvirological Clinical Outcomes

**Figure 6.**
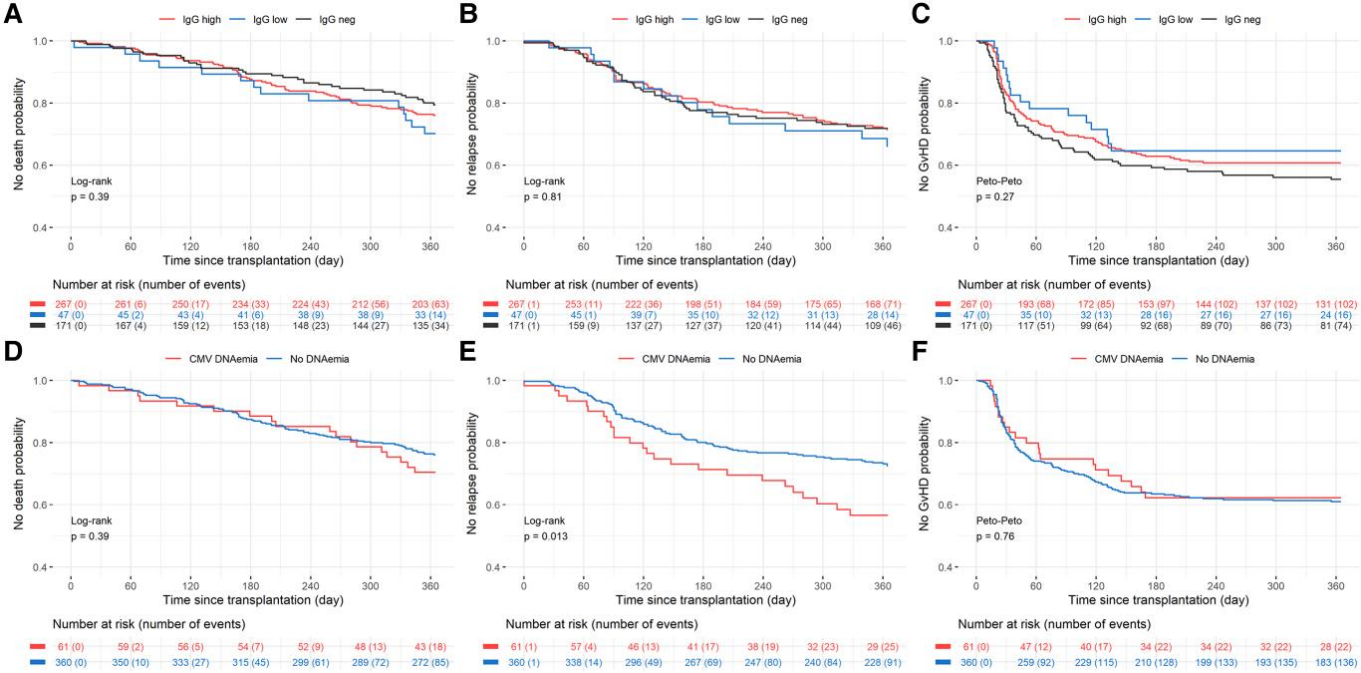
Nonvirological posttransplant clinical outcomes stratified by pretransplant CMV-IgG-titers (*A–C*) or pretransplant CMV-DNAemia (*D–F*) in the whole cohort. Pretransplant CMV-IgG-titers have been classified as high (n = 267), low (n = 47) or negative (n = 171).

Patients with high pretransplant CMV-IgG-titers did not have higher rates of 1-year all-cause mortality, hematologic malignancy relapse, and acute GvHD ≥ grade 2 incidence in the overall population (Figure 6*A*-*C*). Although pretransplant CMV-DNAemia was not associated with all-cause mortality and acute GvHD ≥ grade 2 incidence, there was higher incidence of hematologic malignancy relapse by 1-year posttransplant (Log-rank *P* < .01) (Figure 6*D*-*F*). In multivariable Cox regression analysis to identify mortality predictors, letermovir administration reduced 1-year all-cause mortality (aHR: 0.54, 95% CI: .31–.92, *P*:.02) (Figure 5*B*). In addition, age (aHR:1.03, 95% CI: 1.01–1.05, *P*:.003), HLA-mismatched donor (aHR:1.36, 95% CI: 1.07–1.73, *P*:.01), high pre-HCT relapse score (aHR:2.49, 95% CI: 1.82–3.42, *P* < .001), but also high pre-HCT CMV-IgG-titers (aHR:1.80, 95% CI: 1.10–2.94, *P*:.02) were significant predictors of mortality.

## DISCUSSION

In this single-center cohort study expanding over 8 years, we demonstrate that pretransplant high CMV-IgG-titers or CMV-DNAemia may predict posttransplant CMV virological outcomes, albeit with limited associations with nonvirological clinical outcomes. Several groups have previously reported that pretransplant CMV-IgG-titers may have a potential association with posttransplant CMV reactivation [10, 14–16]. Those studies suffered from large variability of CMV-IgG assays with various cutoffs used and different definitions of CMV outcomes [10]. In addition, none of those studies has reviewed in detail the pretransplant CMV serology status. Based on our institutional procedures and as previously described, 32% of initially considered CMVR + were reclassified as R- [12, 13]. We used a cutoff of CMV-IgG-titers to define low and high IgG titers at 40 IU/mL, based on the association we found between posttransplant csCMVi and pretransplant IgG titers >40 IU/mL. Considering the large span of the study period, several CMV qPCR assays were used and CMV-DNAemia cutoffs to define csCMVi and initiate preemptive CMV treatment changed. To avoid potential biases we included 3 different CMV virological outcomes to describe the overall CMV burden. Regardless of the CMV variable used, we observed a significant association of high pretransplant CMV-IgG-titers with the posttransplant CMV global burden, either defined as csCMVi, any (detectable/quantifiable) CMV-DNAemia, or the number of positive CMV qPCR tests. Furthermore, Cox regression analysis demonstrated high CMV-IgG-titers as the most important independent predictor of posttransplant csCMVi. This suggests a potential association between the magnitude of CMV-IgG-titers and CMV activity of the latent reservoir, although there were no higher rates of posttransplant csCMVi with higher (>40 IU/mL) pretransplant CMV-IgG-titers. On the other hand, it is likely that part of those observations may be the result of selection bias, with a proportion of patients in the low CMV-IgG titer group representing in fact CMVR-. Notably, amongst 47 patients with low CMV-IgG-titers, there were 9 (19%) patients, for whom prior data were not available and who could have potentially been mislabeled as R + . In additional sensitivity analyses performed (data not shown), results remained similar after excluding those 9 patients. Regardless, those data underscore the importance of accurately assessing CMV serology status of allo-HCT recipients pretransplant [12].

Zamora *et al*. reported on the association between pre- and posttransplant CMV replication in a monocentric 10-year retrospective study [8]. Pretransplant CMV-DNAemia in allo-HCT recipients is infrequently performed and limited data are available [8]. We found a prevalence of pretransplant CMV-DNAemia similar to what was previously reported, in the range of 11–12.5% [8]. Pretransplant detectable or quantifiable versus nondetectable CMV-DNAemia was associated with a 2-fold increase in the posttransplant cumulative incidence of both csCMV infections (56% vs 26%) and CMV-DNAemia (92% vs 50%). In addition, pretransplant CMV-DNAemia was identified as an independent predictor of csCMVi. The clinical significance of pretransplant CMV-DNAemia remains to be defined, but might indicate substantial CMV presence in tissues already before transplantation and represent an additional tool in the assessment of pretransplant risk for posttransplant CMV infection. Specific data on pretransplant CMV-DNAemia, including patient population, qPCR values, and preemptive treatment and duration, were not included in this study. Furthermore, our data are further limited by the fact that there were no institutional specific guidelines for the management of pre-HCT CMV-DNAemia, which remained consultant dependent during the study period.

In an effort to assess overall pretransplant CMV burden and define a CMV high-risk profile of patients, we combined both variables, namely pretransplant CMV-IgG-titers and DNAemia. The 6-month cumulative incidence of csCMVi was even higher in patients with both high CMV-IgG and detectable DNAemia (62%), in contrast to patients with low CMV-IgG and undetectable DNAemia who had a relatively low rate of infection (20%). Our data suggest that patients with low pretransplant CMV-IgG-titers and undetectable DNAemia remain at low risk for posttransplant CMV infection, while patients with high IgG titers and CMV-DNAemia pretransplant represent the highest risk group.

The correlation of pretransplant CMV-IgG-titers and DNAemia with posttransplant CMV infection was maintained in the letermovir era, despite the relatively low number of patients included and the significantly lower rates of csCMVi. This suggests that regardless the important reduction of csCMVi associated with the use of letermovir, pretransplant CMV viral reservoir remains an important factor associated with posttransplant CMV reactivation, which needs to be further studied, along with the CMV D/R serology constellation and the posttransplant CMV CMI [6, 17]. In an era when increasing healthcare costs pose major threats, considering the high economical burden of letermovir prophylaxis particularly in low-income countries, and the exposure of large patient populations to universal letermovir prophylaxis, it is pertinent to identify patients that would benefit the most from such an approach.

We also found an association between high CMV-IgG-titers and 1-year mortality, which might be related to direct or indirect consequences of higher rates of csCMVi. In addition, a potential association with disease relapse in patients with pretransplant CMV-DNAemia was found. We hypothesized that this might be, in part, the result of more patients with lymphopenia and lymphoid malignancies and higher HCT comorbidity index included in the pretransplant CMV-DNAemia group, as previously reported [8]. Detailed data on the above mentioned variables were not available, hence no additional conclusions could be made (dedicated manuscript under preparation). We performed additional post hoc correlation analyses between pretransplant CMV-DNAemia and all other host and baseline HCT variables, without any significant associations identified (data not shown). Clearly, more data are required to better describe this observation.

Our study has several limitations, including its retrospective, single-center nature and the comparison of patients from different periods, although results were comparable in the 2 eras despite potential differences in HCT management and epidemiologies. For a number of patients, CMV PCR tests were not performed at pretransplant TID consultation, mostly in the postletermovir period, probably due to outpatient consultations limitation during COVID-19 pandemic. As mentioned above, it is possible that a proportion of low-positive IgG titers might represent passive immunity from prior transfusions and hence misclassification of CMVR- patients as R + . However, considering the low number of those cases, it might have not significantly affected the observed outcomes. Our findings may not be applicable in centers where laboratories report CMV serology results as a dichotomous categorical variable. Finally, measuring CMV-specific CMI is not performed in our center. Data on immunosuppression discontinuation were not available, and hence we were not able to assess the impact of immunosuppression on humoral immunity and late CMV infections.

To conclude, we report significant associations between pretransplant high CMV-IgG-titers and detectable CMV-DNAemia, and posttransplant CMV infections, also in the letermovir prophylaxis era. The clinical significance of the above parameters and how they could be combined with CMV CMI and other host variables to stratify patients to the administration and duration of primary CMV prophylaxis with letermovir requires further investigation. Nevertheless, and while pending further validation, those results could contribute to the careful patient stratification for CMV reactivation, based on the use of simple laboratory tests, such as CMV IgG serology, something which could be of particular value in resource-limited settings.

## Supplementary Material

ofag068_Supplementary_Data

## Supplementary Data

Supplementary materials are available at *Open Forum Infectious Diseases* online. Consisting of data provided by the authors to benefit the reader, the posted materials are not copyedited and are the sole responsibility of the authors, so questions or comments should be addressed to the corresponding author.
