# Supplementary material for: Pretransplant CMV-IgG Titers and DNAemia Are Associated With CMV Infections Post Allogeneic Hematopoietic Cell Transplant With or Without Letermovir
Source: Open Forum Infect Dis. 2026 Feb 16;13(2):ofag068. doi: 10.1093/ofid/ofag068 (PMC12951073; doi:10.1093/ofid/ofag068)
Supplement: ofag068_Supplementary_Data [file ofag068_supplementary_data.docx]

**Supplementary Figure 1. Flow Chart.** HCT: hematopoietic cell transplantation. LET: letermovir. R: recipient.**
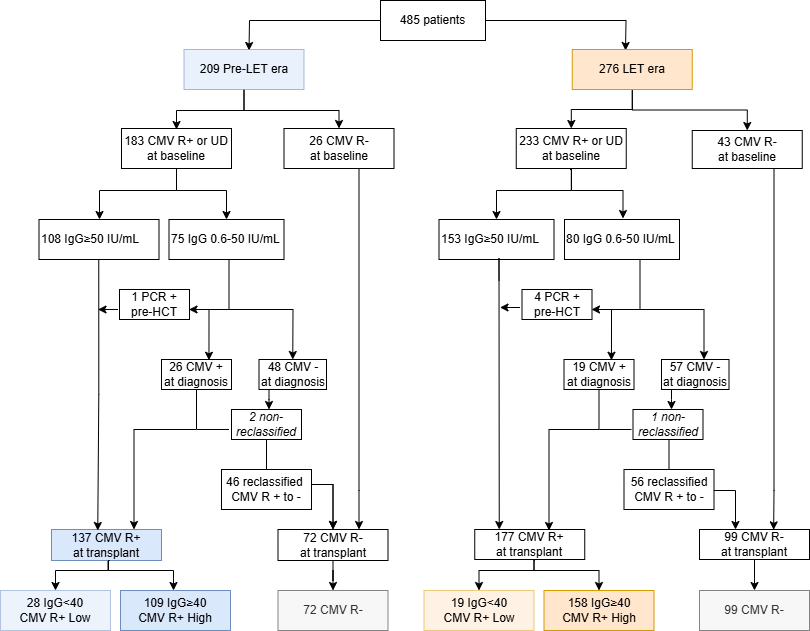
**

**Supplementary Table 1.** Detailed cumulative incidences for clinically significant CMV inrection and CMV DNAemia by day 180 posttransplant.

|  | Overall (95% CI) | Pre-letermovir (95% CI) | Post-letermovir (95% CI) |
| --- | --- | --- | --- |
| First csCMV infection |  |  |  |
| Pre-HCT CMV-IgG titers |  |  |  |
| High | 47.6% (41.4-53.6) | 74.1% (64.5-81.5) | 29.8% (22.7-37.1) |
| Low | 22.5% (11.5-35.7) | 25.3% (10.9-42.6) | 17.5% (4.0-38.8) |
| Negative | 3.6% (1.5-7.2) | 2.8% (0.05-8.8) | 4.1% (1.3-9.5) |
| *p*-value | <0.001 | <0.001 | <0.001 |
| Pre-HCT CMV-DNAemia |  |  |  |
| Detectable | 61.3% (47.6-72.4) | 80.0% (56.7-91.6) | 48.2% (30.8-63.7) |
| Undetectable | 26.9% (22.3-31.6) | 37.8% (30.7-44.9) | 15.9% (10.8-21.8) |
| *p*-value | <0.001 | <0.001 | <0.001 |
| Combined CMV-IgG / CMV-DNAemia |  |  |  |
| High IgG/DT DNAemia | 64.4% (49.6-75.7) | 83.3% (59.2-79.3) | 49.6% (30.5-66.1) |
| High IgG/UD DNAemia | 43.0% (36.22-49.5) | 70.6% (59.4-79.3) | 25.5% (18.3-33.1) |
| Low IgG/UD DNAemia | 22.9% (11.2-37.1) | 26.3% (11.4-44.0) | 15.6% (2.2-40.5) |
| Neg IgG/UD DNAemia | 3% (1.1-6.5) | 2.8% (0.5-8.8) | 3.1% (0.8-8.1) |
| *p*-value | <0.001 | <0.001 | <0.001 |
| First detected CMV DNAemia |  |  |  |
| Pre-HCT CMV-IgG titers |  |  |  |
| High | 87.8% (83.0-91.3) | 96.9% (89.9-99.1) | 81.9% (74.7-87.2) |
| Low | 48.3% (33.0-62.0) | 35.7% (18.5-53.4) | 68.3% (38.9-85.7) |
| Negative | 6.0% (3.0-10.3) | 2.8% (0.5-8.8) | 8.3% (3.8-14.8) |
| *p*-value | <0.001 | <0.001 | <0.001 |
| Pre-HCT CMV-DNAemia |  |  |  |
| Detectable | 91.8% (80.6-96.7) | 96.0% (59.8-99.7) | 88.9% (71.4-96.0) |
| Undetectable | 50.8% (45.4-55.9) | 50.4% (428-57.5) | 51.1% (43.3-58.3) |
| *p*-value | <0.001 | <0.001 | <0.001 |
| Combined CMV-IgG / CMV-DNAemia |  |  |  |
| High IgG/DT DNAemia | 92.7% (80.6-97.4) | 95.8% (58.5-99.7) | 90.3% (70.3-97.1) |
| High IgG/UD DNAemia | 86.4% (80.9-70.5) | 97.3% (88.1-99.4) | 79.9% (71.7-85.9) |
| Low IgG/UD DNAemia | 44.4% (28.6-59.1) | 33.3% (16.4-51.3) | 66.7% (32.1-86.5) |
| Neg IgG/UD DNAemia | 5.4% (2.7-9.6) | 2.8% (0.5-8.8) | 7.3% (3.2-13.7) |
| *p*-value | <0.001 | <0.001 | <0.001 |

csCMV: clinically significant CMV. HCT: hematopoietic cell transplantation. DT: detectable. UD: undetectable. Neg: negative.
